# Use of Bilayer Gate Insulator in GaN-on-Si Vertical Trench MOSFETs: Impact on Performance and Reliability

**DOI:** 10.3390/ma13214740

**Published:** 2020-10-23

**Authors:** Kalparupa Mukherjee, Carlo De Santi, Matteo Borga, Shuzhen You, Karen Geens, Benoit Bakeroot, Stefaan Decoutere, Gaudenzio Meneghesso, Enrico Zanoni, Matteo Meneghini

**Affiliations:** 1 Department of Information Engineering, University of Padua, 35131 Padua, Italy; desantic@dei.unipd.it (C.D.S.); gauss@dei.unipd.it (G.M.); zanoni@dei.unipd.it (E.Z.); matteo.meneghini@unipd.it (M.M.); 2 IMEC, Kapeldreef 75, 3001 Leuven, Belgium; matteo.borga@imec.be (M.B.); shuzhen.you@imec.be (S.Y.); karen.geens@imec.be (K.G.); stefaan.decoutere@imec.be (S.D.); 3 CMST imec/UGent, 9052 Ghent, Belgium; benoit.bakeroot@imec.be

**Keywords:** GaN, vertical GaN, trench MOS, gate dielectric, breakdown, trapping, reliability

## Abstract

We propose to use a bilayer insulator (2.5 nm Al_2_O_3_ + 35 nm SiO2) as an alternative to a conventional uni-layer Al_2_O_3_ (35 nm), for improving the performance and the reliability of GaN-on-Si semi vertical trench MOSFETs. This analysis has been performed on a test vehicle structure for module development, which has a limited OFF-state performance. We demonstrate that devices with the bilayer dielectric present superior reliability characteristics than those with the uni-layer, including: (i) gate leakage two-orders of magnitude lower; (ii) 11 V higher off-state drain breakdown voltage; and (iii) 18 V higher gate-source breakdown voltage. From Weibull slope extractions, the uni-layer shows an extrinsic failure, while the bilayer presents a wear-out mechanism. Extended reliability tests investigate the degradation process, and hot-spots are identified through electroluminescence microscopy. TCAD simulations, in good agreement with measurements, reflect electric field distribution near breakdown for gate and drain stresses, demonstrating a higher electric field during positive gate stress. Furthermore, DC capability of the bilayer and unilayer insulators are found to be comparable for same bias points. Finally, comparison of trapping processes through double pulsed and V_th_ transient methods confirms that the V_th_ shifts are similar, despite the additional interface present in the bilayer devices.

## 1. Introduction

Vertical GaN technologies [1,2,3,4,5,6,7,8,9,10] are gaining popularity for power conversion applications [8,11], owing to the superior power handling capabilities compared to the lateral configuration [12,13,14], combined with the inherent material advantages of GaN. Among various vertical architectures, trench MOSFETs on Si substrates [3,4,8,10] have substantial economic advantages, in addition to promising performance metrics. However, development is still in the initial stages, and active research into the MOS framework is ongoing.

The choice and quality of the dielectric is an important factor in determining device stability and reliability. However, the performance and degradation issues related to the gate oxide of vertical GaN MOSFETs still have to be investigated in detail. Moreover, several papers (see for instance [15,16]) propose the use of a single dielectric (typically Al_2_O_3_) as a gate insulator; alternative solutions, such as the use of bi-layered dielectrics, still have to be explored: bilayer dielectrics are used in silicon MOSFETs, and typically consist of a thin interface dielectric followed by a thicker insulator.

The aim of this work is to advance the understanding on the leakage, reliability, and trapping related to the gate stack of vertical GaN-on-Si MOSFETs. Specifically, (i) we investigate the degradation processes of the gate insulator subject to electrical stress at high field for devices with uni-layer dielectrics; (ii) we propose to use a bilayer dielectric stack (Al_2_O_3_ followed by SiO_2_) with the aim of improving device stability and reliability; (iii) we demonstrate the net superiority of the bilayer dielectric scheme, compared to the uni-layer. The results described in this paper indicate that bilayer dielectrics are an advantageous alternative to uni-layer ones for the fabrication of vertical GaN MOSFETs, and provide general guidelines for device design.

It was chosen to keep a thin interfacial layer of Al_2_O_3_ on top of the AlGaN, since this allows for substantially improving the quality of the interface, compared to a SiO_2_/GaN interface (see recent reports on the topic, such as, for instance, [17,18]). The SiO_2_ layer was introduced in order to improve the breakdown strength of the devices, compared to the case in which Al_2_O_3_ alone is used. The article is structured as follows: in Section 2, the structural details of the studied semi-vertical devices, and of the simulated structure are described. Section 3 compares DC and breakdown tests, which show a reduction of gate leakage by two orders of magnitude with bilayer devices, while ensuring comparable drain currents in the on-state. Section 4 summarizes the observations and concludes the work. The bi-layer approach is found to enhance breakdown robustness, based on observations from step stress and constant voltage stress experiments. Finally, the comparison of V_th_ shifts under positive gate stress reveals that the use of the bilayer dielectric does not notably deteriorate trapping processes in the devices.

## 2. Materials and Methods 

**Figure 1:**
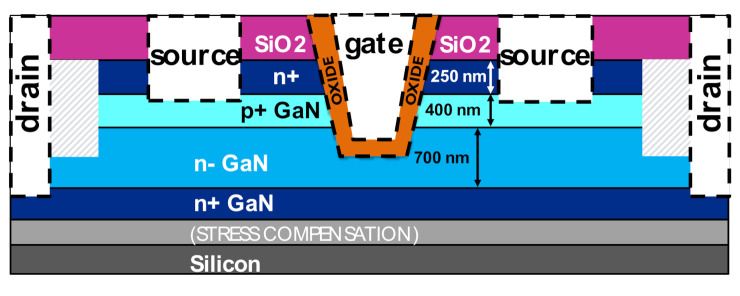
Structural schematic of the studied semi vertical trench MOSFETs.

A semi-vertical device configuration is used as a test vehicle to investigate dielectric processing, in gate trench MOSFETs for vertical GaN devices, as described in the schematic in Figure 1. The devices were fabricated on a 200 mm silicon substrate.

The bottom of the gate trench is 4 µm in length. The width of each gate finger is 100 µm. The gate trench extends into the n-drift layer. The structure is semi-vertical, meaning that electrons flow from the source, vertically along the gate trench and n-drift region, and then laterally through the buried n+ layer to the drain via, which finally returns the current to the surface through the drain metal. Two wafers with varying gate dielectric from IMEC, Leuven, Belgium are compared: Wafer M with a 35 nm uni-layer aluminum oxide (Al_2_O_3_) and Wafer B with a bilayer composition: 2.5 nm Al_2_O_3_ and 35 nm silicon dioxide (SiO_2_). In both wafers, the Al_2_O_3_ layer has been deposited, using atomic layer deposition (ALD) at 300 °C. For the SiO_2_ in the bi-layer, a plasma enhanced chemical vapor deposition (PECVD) oxide was used with a deposition temperature of 400 °C.

The Sentaurus tool from Synopsys was used for the TCAD simulations in order to investigate the electric field distribution.

## 3. Results and Discussion

### 3.1. DC Parameters and Characterization

**Figure 2:**
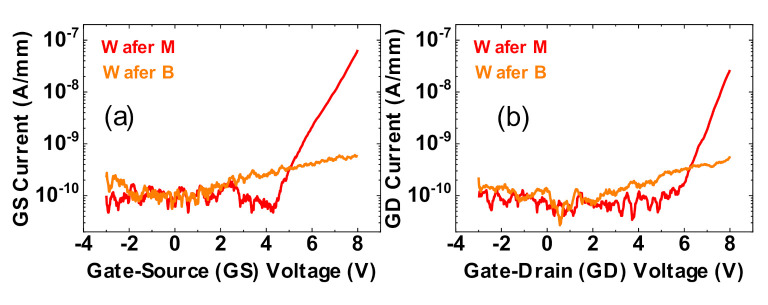
Comparison of diode leakage behavior between Wafer M (uni-layer oxide) and Wafer B (bilayer oxide). (**a**,**b**) are typical characteristics of the gate-source (GS) diode with drain floating (**a**) and the gate-drain (GD) diode with source floating (**b**).

**Figure 3:**
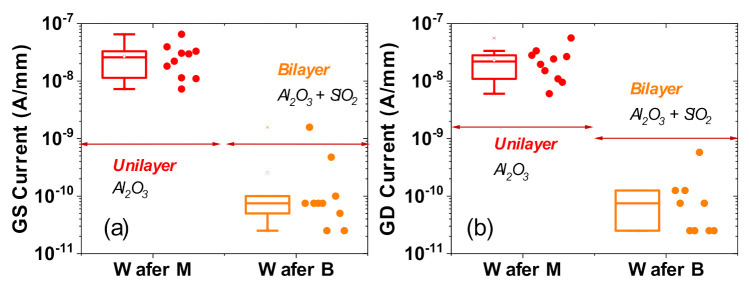
Comparison of diode leakage behavior between Wafer M (uni-layer oxide) and Wafer B (bilayer oxide). (**a**,**b**) show statistical results of the current level at V_GS_ = 8 V (**a**) and V_GD_ = 8 V (**b**).

Figure 2a,b present typical examples of the gate-source and gate-drain leakage characteristics, respectively, for both wafers, for V_GS_ (with the drain terminal floating) and V_GD_ (with the source terminal floating) sweeps from −3 V to +8 V. Figure 3a,b summarize the leakage distribution at V_GS_ = 8 V and V_GD_ = 8 V, respectively, over several tested devices from each wafer.

With Wafer B (bilayer wafer), the gate leakage is clearly lower, by more than two orders of magnitude, compared to Wafer M (uni-layer wafer). This difference may be ascribed to the intrinsic properties of the material, like the critical breakdown field, which is lower in Al_2_O_3_, compared to SiO_2_.

**Figure 4:**
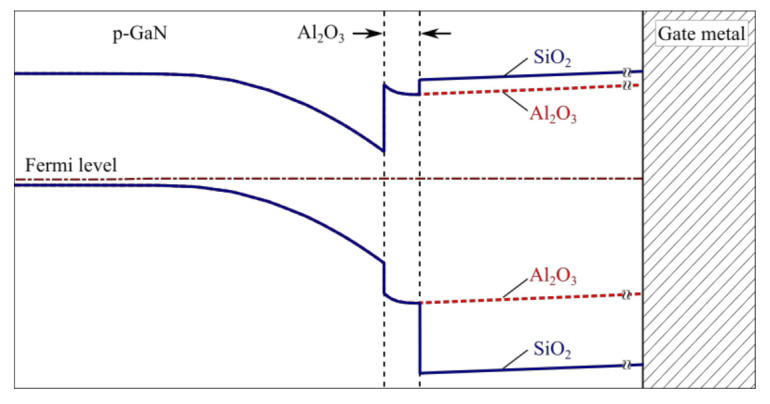
Schematic of the conduction band discontinuity at the Al_2_O_3_/SiO_2_ interface within the bilayer dielectric stack, in comparison to the Al_2_O_3_ only uni-layer stack.

A second factor to take into account is that the use of the bilayer increases the barrier for thermionic electron leakage from the channel to the metal (as shown in Figure 4). By adding SiO_2_ to the gate oxide stack, the effective barrier respective to the gate metal is increased.

An additional conduction band discontinuity of 0.4 eV is present at the interface between Al_2_O_3_ and SiO_2_ [19], which contributes to an effectively higher band offset with GaN, relative to the gate. Thus, an associated reduction of the leakage current is expected, which is especially relevant at high positive gate biases.

**Figure 5:**
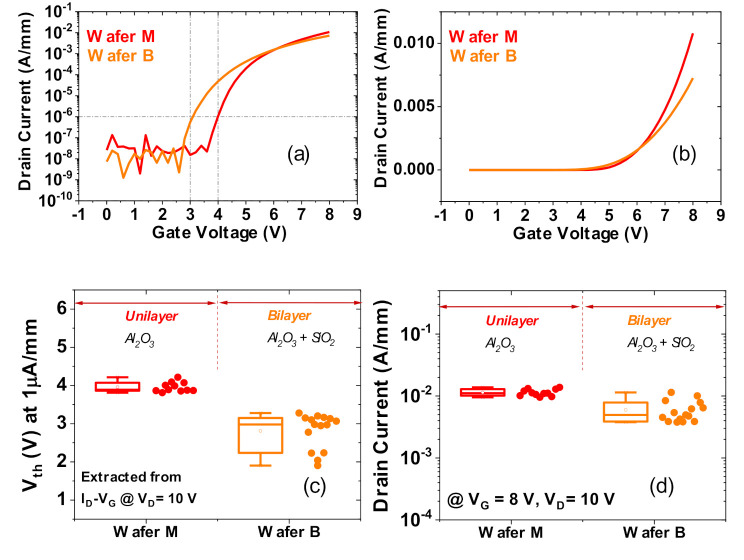
Comparison of transfer characteristics between Wafer M (uni-layer oxide) and Wafer B (bilayer oxide). Typical I_D_-V_GS_ results at V_DS_ = 10 V in (**a**) log scale and (**b**) linear scale. (**c**,**d**) box charts with statistics of threshold voltage (V_th_) (**c**) and I_DS_ current levels at V_GS_ = 8 V and V_DS_ = 10 V (**d**).

Figure 5a,b illustrates a typical I_D_-V_GS_ characteristic from each wafer at V_DS_ = 10 V in log scale and linear scale, respectively. From measurements on several devices, the threshold voltage (V_th_) is extracted at 1μA/mm, and the associated distribution is presented in Figure 5c. The drain current distribution at V_GS_ = 8 V and V_DS_ = 10 V is compiled in Figure 5d for both wafers.

The mean threshold voltage is found to be ≈4 V for Wafer M and ≈3 V for Wafer B. Importantly, from the I_DS_ comparison, it can be observed that, for identical bias conditions, the drain current level is comparable between the two cases. Based on the results described within this section, we can conclude that the use of a bilayer passivation substantially improves the leakage performance of the devices, without deteriorating the DC on-state current.

### 3.2. Drain-Gate Step Stress Experiment

The results described above indicate that a bilayer insulator permits obtaining a substantial reduction in the gate leakage, for comparable DC performance of the transistors. In this section, we evaluate the robustness of devices with uni-layer and bilayer insulators. To this aim, 35 devices from each wafer were subjected to off-state drain step stress tests at V_GS_ = 0 V. The drain stress (V_DS_) bias was incremented in steps of 3 V until breakdown with 120 s per step.

**Figure 6:**
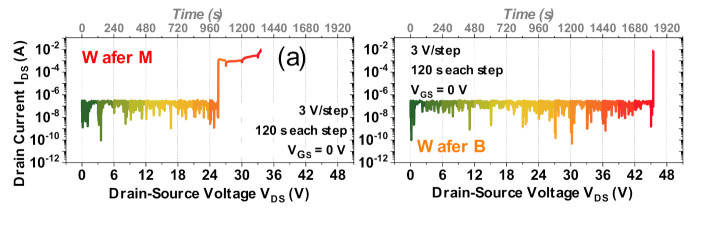

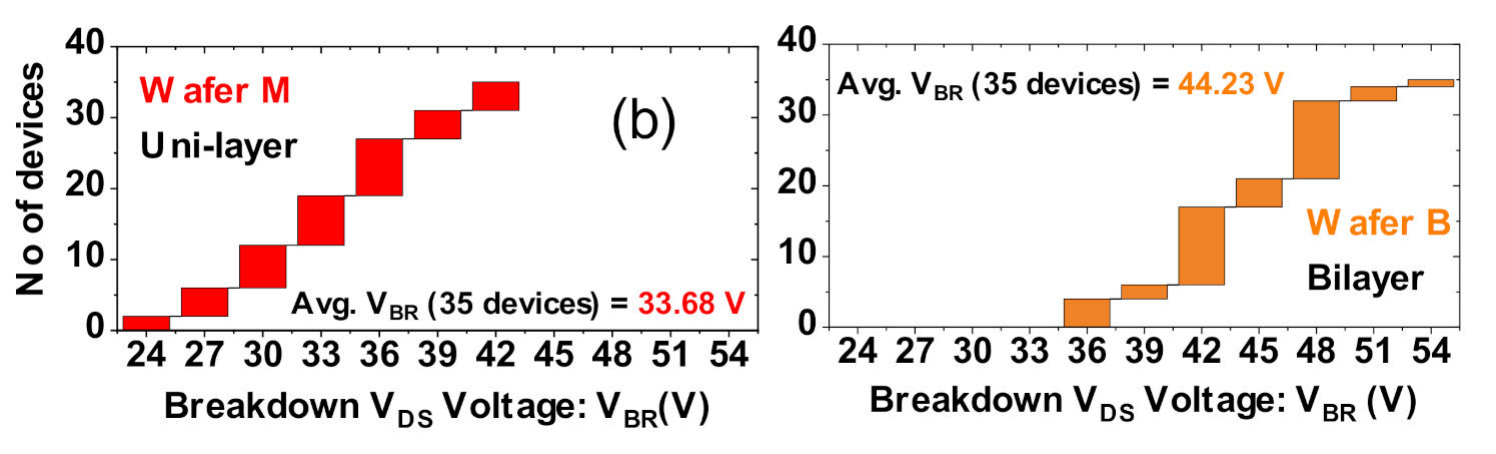
Off-state drain step stress comparison at V_GS_ = 0 V for Wafer M (uni-layer) and Wafer B (bilayer) devices. (**a**) drain-source current (I_DS_) evolution during stress; (**b**) breakdown drain-source voltage (V_BR_) distribution for 35 tested devices from each wafer.

Figure 6a reports representative examples of the I_DS_ evolution during the step stress test, for each of the wafers. As can be noticed, drain leakage remains stable and low (around 100 nA) until a breakdown is observed. Failure consists of a sudden increase in leakage current, which is indicative of catastrophic breakdown in the insulating layer. I_GS_ evolution (not shown) is found to be similar in nature, indicating that leakage current is dominated by the gate–drain component.

Figure 6b reports the cumulative failure distributions for the two wafers; as can be noticed, the mean breakdown voltage for Wafer B (with an average = 44.2 V) is observed to be more than 10 V higher than Wafer M (with an average = 33.7 V).

This represents a relevant reliability advancement for the wafer with bilayer insulator, considering that the thickness of the dielectric is only 2.5 nm thicker than Wafer M. This difference can be ascribed to the different breakdown strengths of the two insulators. Silicon dioxide has a much lower dielectric constant (3.9, [20]) compared to aluminum oxide (9.1, [20]). It is known that the breakdown field decreases with increasing dielectric constant [21,22]. Therefore, silicon dioxide has a higher breakdown field, compared to aluminum oxide.

### 3.3. Reliability: Electroluminescence Tests

Step stress tests on the drain were also performed in conjunction with electroluminescence imaging to investigate the nature and location of the breakdown spot.

During a step–stress test, typically the samples are destroyed, due to a sudden increase in leakage current. However, catastrophic failure should be prevented, if one wants to use the samples for failure analysis. To this aim, we carried out the step–stress tests by using a current limiting circuit that was added to the microprobes. Once the device reaches breakdown, this circuit instantly decreases the voltage on the devices to avoid thermal runaway at the failure location. This allows for keeping the samples in good status, thus permitting post-stress electroluminescence characterization, to accurately identify the failure spot.

**Figure 7:**
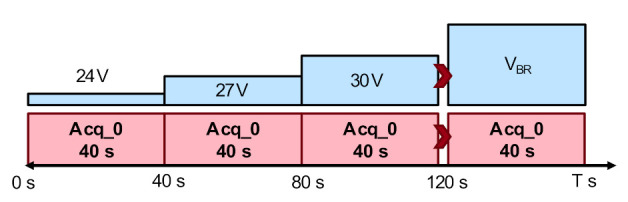
Electroluminescence imaging procedure coupled with step stress.

We tested a total of 31 devices. As displayed in Figure 7, stress voltage (V_DS_) was set at 24 V, and increased by 3 V every 40 s, until failure was reached. A 40 s EL image was simultaneously acquired at each V_DS_. 

**Figure 8:**
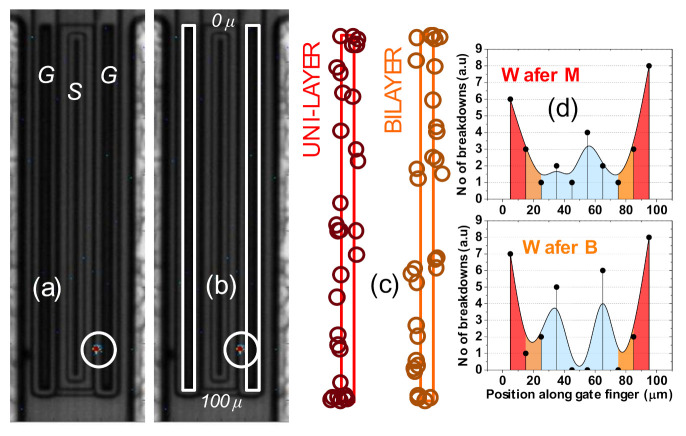
Breakdown spot mapping using V_DS_ step stress with electroluminescence imaging (**a**) example of a device image with an observed breakdown spot; (**b**) outline of the two 100 μm gate fingers which together represent the map area; (**c**) EL spot locations over both gate fingers superimposed onto the mapping region, for each wafer (**d**) number of breakdown occurrences distributed over 10 equal intervals along the width of the gate fingers.

Once breakdown is achieved, a single post-breakdown stress (with an EL acquisition of 40 s) at the corresponding V_BR_ is performed to verify the location of the detected luminescence spots. The EL spot measured at V_BR_ reflects the region of breakdown in the devices. Figure 8a displays a typical EL device image obtained at V_DS_ = V_BR_, marking the breakdown location. The rectangular mapping region for each wafer was obtained by considering both gate fingers (see Figure 8b) concurrently. In Figure 8c, the EL spot positions along the gate fingers were collated for each wafer, to acquire the spatial distribution of the breakdown occurrences per 10 μm of the mapped region, as illustrated in Figure 8d.

From the distribution in Figure 8d, it can be inferred that the breakdown occurs preferentially near the corners of the gate fingers (top and bottom 10 μm regions within the 100 μm fingers). For both wafers, almost 50% of tested devices (8 + 6 out of 31 for Wafer M and 8 + 7 out of 31 for Wafer B) show breakdown spots at the gate edges. Hence, breakdown location is found to be independent of the dielectric composition.

### 3.4. Reliability: GS/GD Breakdown and TCAD Simulations

Multiple devices were subjected to forward gate step stress tests at V_DS_ = 1 V to evaluate the maximum voltage applicable to the gate of the devices, and to compare the gate-source breakdown characteristic for the wafers. The gate was biased in 3 V steps of 120 s each, until breakdown was reached.

**Figure 9:**
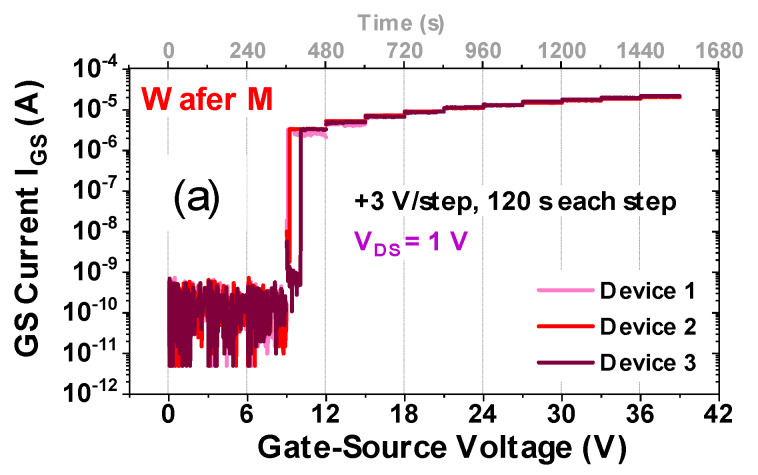

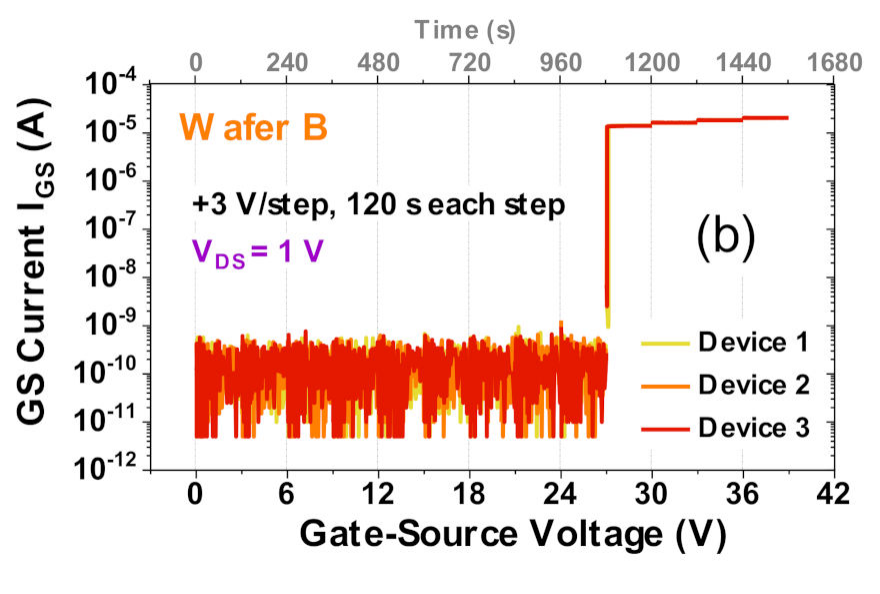
Forward gate-source step stress at V_DS_= 1 V. (**a**,**b**) present typical stress current (I_GS_) characteristics for three Wafer M (uni-layer) devices (**a**) and three Wafer B (bilayer) devices (**b**).

Electrical measurements were coupled with electroluminescence studies, in order to obtain a detailed description of the degradation process. It is observed here as well that the failure is focused around the gate corners (not shown). The I_GS_ evolution for three representative devices from each wafer is presented in Figure 9a,b, respectively.

It is clear that the bilayer devices are consistently more robust than uni-layer devices, displaying an average gate breakdown voltage of 27 V, which is substantially higher than the gate breakdown around 9 V observed for the Wafer M devices. This represents an important improvement in gate reliability.

**Figure 10:**
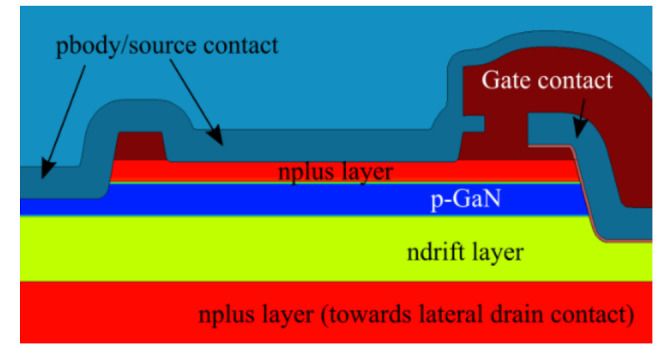
Simulated structure representing the GaN trench MOSFET devices.

The gate-source step stress experiment probes the devices under on-state conditions, compared to the off state drain-source step stress results discussed above. Accordingly, breakdown voltages obtained for V_GS_ stress are correspondingly lower for both wafers. (44 V to 27 V for Wafer B, and 33 V to 9 V for Wafer M). TCAD simulations can be used to visualize the region near the gate under both of these conditions. The representative structure used to simulate the devices is presented in Figure 10.

**Figure 11:**
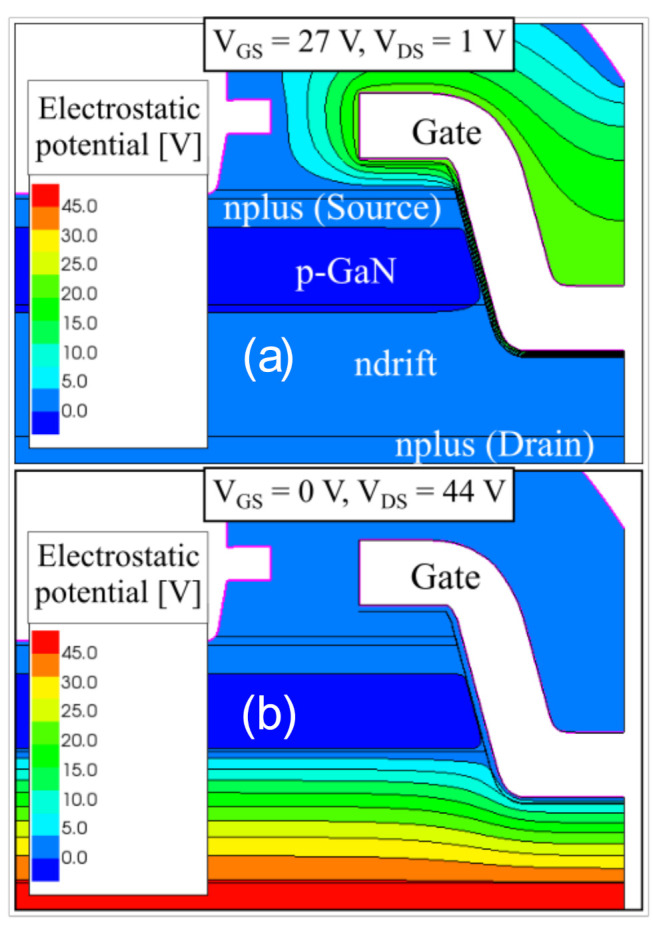
Simulations of breakdown conditions for the bilayer devices: (**a**) in the ON-state with V_GS_ = 27 V and V_DS_ = 1 V and (**b**) in the OFF-state with V_GS_ = 0 V and V_DS_ = 44 V.

Figure 11 displays the equipotential lines around the gate of the bilayer device structure: (a) for a gate-source voltage sweep at V_GS_ = 27 V and V_DS_ = 1 V and (b) for a drain-source voltage sweep under OFF-state conditions at V_DS_ = 44 V and V_GS_ = 0 V.

For a positive V_DS_ sweep under OFF-state, the channel is not formed, and the potential drops on both the oxide and the depleted drift region. From Figure 11b, we can observe the increase of the electric field below the trench within the n-drift layer. The associated depletion within the drift region absorbs part of the applied voltage. Hence, the critical electric field inside the gate dielectric is obtained at higher drain voltages compared to the ON-state.

Indeed, in the ON-state (forward gate bias), the channel is formed along the gate trench sidewalls. As such, as can be seen from Figure 11a, the applied gate voltage falls almost entirely across the oxide and the critical electric field in the gate dielectric is reached at lower gate bias voltages. Hence, the device breakdown voltage under forward gate stress provides a better representation of the critical electric field associated with a chosen dielectric.

Based on the results of the step–stress experiments, for Wafer M (uni-layer dielectric), a basic critical electric field value of 2.6 MV/cm (9 V/35 nm) can be calculated. For the potential of 27 V across the bilayer dielectric stack, the potential at the boundary between SiO_2_ and Al_2_O_3_ can be calculated to be 0.80 V. This corresponds to an estimated electric field value of 7.5 MV/cm (26.2 V/35 nm) across the SiO_2_ layer, and 3.2 MV/cm (0.80 V/2.5 nm) across the Al_2_O_3_ layer, in Wafer B devices.

Taking these values as reference electric fields for which breakdown can be assumed to occur, simulations were performed on vertical trench MOS structures with uni-layer and bi-layer dielectric configurations to assess expected breakdown voltages under ideal conditions.

**Figure 12:**
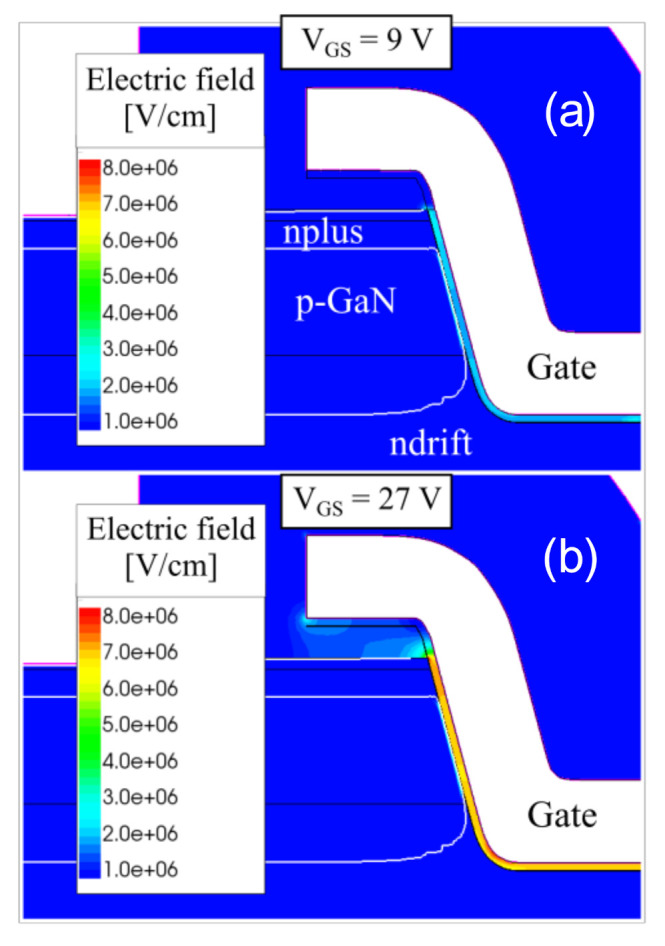
Simulations of the electric field under on-state V_GS_ biasing on Wafer M and Wafer B structures: for the uni-layer device at V_GS_ = 9 V and V_DS_ = 1 V (**a**), for the bilayer device at V_GS_ = 27 V and V_DS_ = 1 V (**b**).

Figure 12 presents the false color maps of the electric field near the gate trench edges; these simulations were done at the breakdown voltage values estimated for the two wafers by step–stress experiments.

Specifically, we used V_GS_ equal to 9 V at V_DS_ = 1 V for the uni-layer and (b) V_GS_ equal to 27 V at V_DS_ = 1 V for the bilayer device structures. There is electric field crowding near the trench edges for both the cases. In Figure 12a, we can see that the electric field value of 2.6 MV/cm estimated from the experiments approximately corresponds to the V_GS_= 9 V condition in the uni-layer device. Similarly, from Figure 12b, at V_GS_ = 27 V in the bilayer device, we observe that the calculated field value of 7.5 MV/cm reasonably matches the simulated electric field peak in the SiO_2_ layer near the gate trench edge. Thus, the simulated gate breakdown voltages closely approach the experimentally obtained gate voltage limits. This is indicative of a reliable dielectric deposition process used here on GaN.

### 3.5. Reliability: Constant Voltage Stress

Constant voltage stresses were performed on 12 devices for different V_DS_ values per wafer to assess the failure probabilities and derive lifetimes from Weibull analysis. For Wafer M, stress voltages were defined at V_DS_ = 28, 30 and 35 V (around the mean V_BR_ of 33.6 V) and for Wafer B at V_DS_ = 42, 45 and 50 V (around the mean V_BR_ of 44 V).

**Figure 13:**
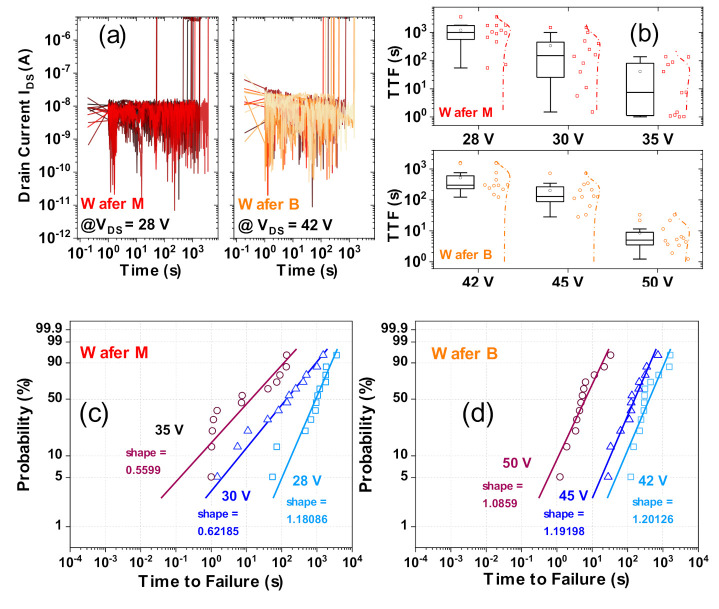
Constant voltage stress tests on Wafer M (uni-layer) and Wafer B (bilayer). (**a**) presents current plots for 12 tested devices at a V_DS_ bias of 28 V for Wafer M and 42 V for Wafer B; (**b**) Time to Failure (TTF) dependence on stress voltage V_DS_ for constant voltage stress tests on both wafers; (**c**,**d**) are probability distributions evaluated for Wafer M (**c**) and Wafer B (**d**) devices.

Based on the constant voltage test results, as displayed for the low voltage biases of 28 V (Wafer M) and 42 V (Wafer B) in Figure 13a, the time to failure (TTF) distribution is summarized in Figure 13b. The Weibull probability percentages are then calculated for both wafers at each voltage and presented in Figure 13c,d for Wafer M and Wafer B, respectively. The plot evaluates the Weibull cumulative probability to determine whether the time to failure under different voltages represents a Weibull distribution [23]. The slope of the Weibull plot (shape factor in Figure 13c,d) reflects the conformity of the obtained fit.

From Figure 13c,d, the constant voltage TTF data appear to reasonably agree with Weibull distribution fits, for both wafers, except for some outliers with very short failure times. The shape factor for Wafer M is below 1 for V_DS_ = 30 V and 35 V. This indicates that, at these higher voltages, early life or extrinsic device failures dominate.

Only at the lowest V_DS_ = 28 V, the shape factor is 1.18, indicating a shift to intrinsic failures, which could be linked to random events or wearout mechanisms. For the bilayer devices (Wafer B), the shape factors for all V_DS_ cases are all close to or above 1 (constant or increasing failure rate with time), which indicates that the device failures are probably due to wearout mechanisms. This confirms the superior stability of the bilayer dielectric, compared to the uni-layer case.

### 3.6. Trapping Performance: Threshold Voltage Shifts

In order to evaluate the impact of using a bilayer dielectric on trapping processes, we investigated the dynamic performance of the two wafers under positive gate stresses.

#### 3.6.1. Double Pulsed Measurements

**Figure 14:**
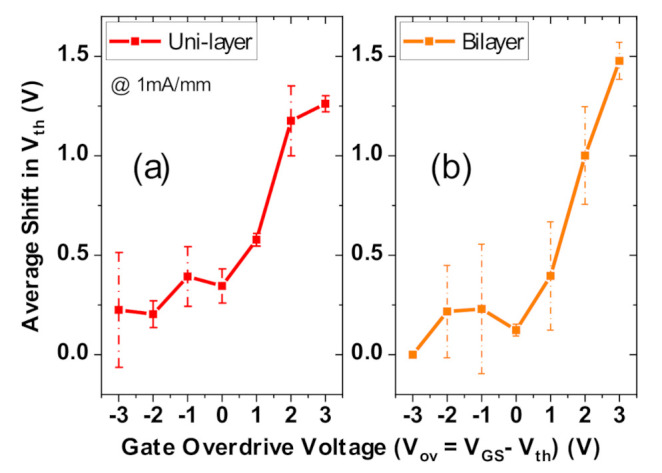
Average V_th_ shift comparison between Wafer M (uni-layer) and Wafer B (bilayer) from double pulsed measurements. (**a**,**b**) are V_th_ shifts for quiescent V_GS_ stress voltages up to gate overdrive = 3V (quiescent V_DS_ = 0 V), for Wafer M with V_th_ ≈ 4V and V_GS,Stress_ = 0 to 7 V (**a**) and for Wafer B with V_th_ ≈ 3 V and V_GS,Stress_ = 0 to 6 V).

Fast double pulsed measurements [24,25] were performed on both wafers for positive gate quiescent voltages up to an overdrive V_ov_ (=V_GS_-V_th_) of 3 V and a quiescent V_DS_ = 0 V. The quiescent periods are interrupted by measurement periods to build an I_DS_–V_GS_ characteristic specific to a particular V_GS,Stress_ (yielding V_th_). The t_Q_ and t_meas_ periods were 100 µs and 1 µs, respectively. The extracted V_th_ shifts at each stress voltage (V_th_ @ V_GS,Stress_ -V_th_ @ 0 V) were averaged from several devices, and then compared in Figure 14a,b, for Wafer M and Wafer B, respectively.

It is observable that the V_th_ shifts for similar overdrive voltages are comparable between the wafers. These results indicate that most of the trapping occurs at interface and/or border traps at the GaN/Al_2_O_3_ interface [25,26,27], which is common to both dielectric configurations.

We notice that the bilayer devices have slightly larger shifts (≈0.2 V), at the highest overdrive of 3 V. This is reasonable to expect since the bilayer structure introduces an additional interface inside the gate dielectric, which could be a source of additional trapping sites.

#### 3.6.2. V_th_ Transient Measurements

**Figure 15:**
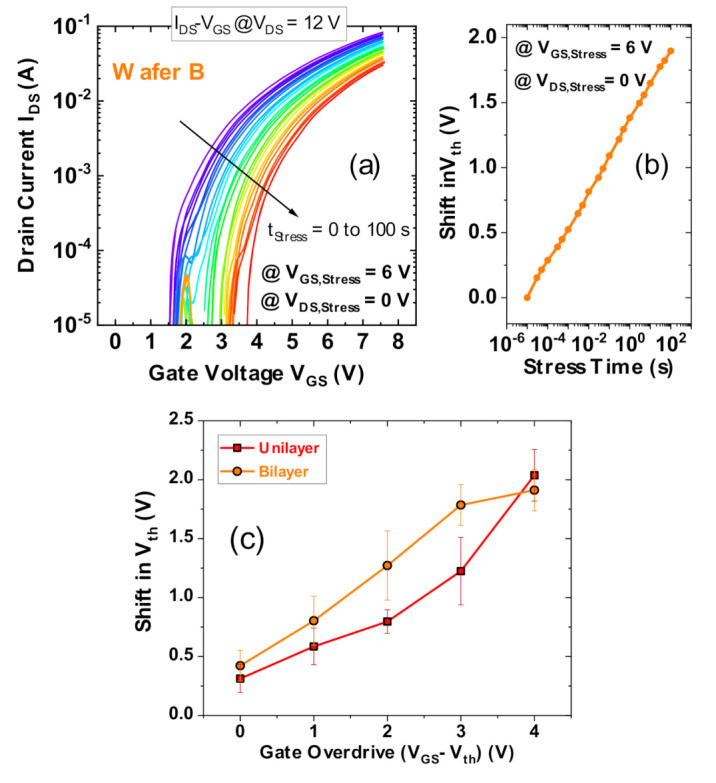
V_th_ shift comparison from V_th_ transient measurements between wafers for V_GS_ stress voltages up to 4 V of overdrive. (**a**) displays a typically obtained set of 22 I_D_–V_GS_ curves during 100 s of stress at V_GS,Stress_ = 6 V and V_DS,Stress_ = 0 V on a Wafer B device; the associated V_th_ shift is plotted in (**b**). Averaged V_th_ shifts (at 100 s) for 6 devices each, are plotted in (**c**) for V_GS,Stress_ = 0 to 7 V for Wafer B with V_th_ ≈ 3 V and V_GS,Stress_ = 0 to 8 V for Wafer M with V_th_ ≈ 4 V.

Finally, the V_th_ transient method [25,28,29] was used to accurately extract the V_th_ shifts for each wafer for V_ov_ up to 4 V for a stress time of 100 s. During each stress phase at a given V_GS,Stress_, 22 fast (10 μs) I_DS_-V_GS_ sweeps (V_GS_ = 0 to 7.5 V at V_DS_ = 12 V) were performed, over a total time duration of 100 s, to measure the gradual shifts in V_th_. Figure 15a illustrates a typical I_DS_–V_GS_ characteristic set at V_GS,Stress_ = 6 V for Wafer B during the stress phase, and Figure 15b presents the extracted V_th_ shift (defined for a current intercept of 2 mA/mm). The V_th_ shifts after 100 s of each V_GS,Stress_ were averaged over six devices from each wafer, and compared in Figure 15c for the same overdrive.

The maximum V_th_ shift is around 2 V for both wafers at the highest overdrive of 4 V. Wafer B has moderately higher V_th_ shifts than Wafer M.

## 4. Conclusions

In summary, we demonstrated that, by using a bilayer insulator, it is possible to improve substantially the performance and the reliability of GaN-based vertical MOSFETs. For almost the same insulator thickness, the devices with bilayer insulator have a gate leakage two orders of magnitude lower, a higher drain breakdown voltage (44.2 V vs. 33.7 V), a higher gate-source breakdown voltage (27 V vs. 9 V). From constant voltage stress tests, the uni-layer shows an extrinsic failure at 30 V (average V_BR_ − 3.7 V), while the bi-layer failures indicate wear out mechanisms even at 50 V (average V_BR_ + 5.8 V), which highlights its better robustness. Electroluminescence studies and simulations were employed to understand the electric field evolution with stress at different stages of the degradation process. In addition to having an improved breakdown robustness for the bilayer dielectric, the trapping effects in terms of V_th_ shifts in both wafers were found to be comparable, which renders the bilayer dielectric configuration highly favorable for use in vertical GaN trench MOS technologies.

## References

[1] Li W., Nomoto K., Lee K., Islam S.M., Hu Z., Zhu M., Gao X., Pilla M., Jena D., Xing H.G. (2018). Development of GaN vertical trench-MOSFET with MBE regrown channel. IEEE Trans. Electron Devices.

[2] Hu J., Zhang Y., Sun M., Piedra D., Chowdhury N., Palacios T. (2018). Materials and processing issues in vertical GaN power electronics. Mater. Sci. Semicond. Process..

[3] Liu C., Khadar R.A., Matioli E. (2017). GaN-on-Si quasi-vertical power MOSFETs. IEEE Electron. Device Lett..

[4] Khadar R.M.A., Liu C., Soleimanzadeh R., Matioli E. (2019). Fully vertical GaN-on-Si power MOSFETs. IEEE Electron Device Lett..

[5] Otake H., Chikamatsu K., Yamaguchi A., Fujishima T., Ohta H. (2008). Vertical GaN-based trench gate metal oxide semiconductor field-effect transistors on GaN bulk substrates. Appl. Phys. Express.

[6] Gupta C., Chan S.H., Lund C., Agarwal A., Koksaldi O.S., Liu J., Enatsu Y., Keller S., Mishra U.K. (2016). Comparing electrical performance of GaN trench-gate MOSFETs with a-plane and m-plane sidewall channels. Appl. Phys. Express.

[7] Khadar R.A., Liu C., Zhang L., Xiang P., Cheng K., Matioli E. (2018). 820-V GaN-on-Si quasi-vertical pin diodes with BFOM of 2.0 GW/cm2. IEEE Electron Device Lett..

[8] Flack T.J., Pushpakaran B.N., Bayne S.B. (2016). GaN technology for power electronic applications: A review. J. Electron. Mater..

[9] Zhang Y., Dadgar A., Palacios T. (2018). Gallium nitride vertical power devices on foreign substrates: A review and outlook. J. Phys. D Appl. Phys..

[10] Liu C., Khadar R.A., Matioli E. 645 V quasi-vertical GaN power transistors on silicon substrates. Proceedings of the 2018 IEEE 30th International Symposium on Power Semiconductor Devices and ICs (ISPSD).

[11] Ma C.T., Gu Z.H. (2019). Review of GaN HEMT applications in power converters over 500 W. Electronics.

[12] De Santi C., Meneghini M., Meneghesso G., Zanoni E. (2017). Review of dynamic effects and reliability of depletion and enhancement GaN HEMTs for power switching applications. IET Power Electron..

[13] Amano H., Baines Y., Beam E., Borga M., Bouchet T., Chalker P.R., Charles M., Chen K.J., Chowdhury N., Chu R. (2018). The 2018 GaN power electronics roadmap. J. Phys. D Appl. Phys..

[14] Ibbetson J.P., Fini P.T., Ness K.D., DenBaars S.P., Speck J.S., Mishra U.K. (2000). Polarization effects, surface states, and the source of electrons in AlGaN/GaN heterostructure field effect transistors. Appl. Phys. Lett..

[15] Ruzzarin M., Meneghini M., Bisi D., Sun M., Palacios T., Meneghesso G., Zanoni E. (2017). Instability of Dynamic-*R_ON_* and Threshold Voltage in GaN-on-GaN Vertical Field-Effect Transistors. IEEE Trans. Electron Devices.

[16] Ruzzarin M., Meneghini M., De Santi C., Meneghesso G., Zanoni E., Sun M., Palacios T. Degradation of Vertical GaN FETs under Gate and Drain Stress. Proceedings of the 2018 IEEE International Reliability Physics Symposium (IRPS).

[17] Gao J., Hao M., Li W., Xu Z., Mandal S., Nemanich R., Chowdhury S. (2018). Al2O3 Insertion Layer for Improved PEALD SiO2/(Al) GaN Interfaces. Phys. Status Solidi A.

[18] Posthuma N.E., You S., Stoffels S., Liang H., Zhao M., Decoutere S. Gate Architecture Design for Enhancement Mode p-GaN Gate HEMTs for 200 and 650V Applications. Proceedings of the 2018 IEEE 30th International Symposium on Power Semiconductor Devices and ICs (ISPSD).

[19] Robertson J., Falabretti B. (2016). Band offsets of high K gate oxides on III-V semiconductors. J. Appl. Phys..

[20] Robertson J. (2004). High dielectric constant oxides. Eur. Phys. J. Appl. Phys..

[21] Jain P., Rymaszewski E.J. (2002). Embedded thin film capacitors-theoretical limits. IEEE Trans. Adv. Packag..

[22] Klee M., De Esch M., Keur W., Kumar B., van Leuken-Peters L., Liu J., Mauczok R., Neumann K., Reimann K., Renders C. (2009). Ferroelectric thin-film capacitors and piezoelectric switches for mobile communication applications. IEEE Trans. Ultrason. Ferroelectr. Freq. Control.

[23] Nelson W. (1980). Accelerated life testing-step-stress models and data analyses. IEEE Trans. Reliab..

[24] Bisi D., Stocco A., Meneghini M., Rampazzo F., Cester A., Meneghesso G., Zanoni E. High-voltage double-pulsed measurement system for GaN-based power HEMTs. Proceedings of the 2014 IEEE International Reliability Physics Symposium.

[25] Mukherjee K., Borga M., Ruzzarin M., De Santi C., Stoffels S., You S., Geens K., Liang H., Decoutere S., Meneghesso G. (2020). Analysis of threshold voltage instabilities in semi-vertical GaN-on-Si FETs. Appl. Phys. Express.

[26] Ren B., Sumiya M., Liao M., Koide Y., Liu X., Shen Y., Sang L. (2018). Interface trap characterization of Al2O3/GaN vertical-type MOS capacitors on GaN substrate with surface treatments. J. Alloys Compd..

[27] Kaneki S., Ohira J., Toiya S., Yatabe Z., Asubar J.T., Hashizume T. (2016). Highly-stable and low-state-density Al2O3/GaN interfaces using epitaxial n-GaN layers grown on free-standing GaN substrates. Appl. Phys. Lett..

[28] Lagger P., Reiner M., Pogany D., Ostermaier C. (2014). Comprehensive study of the complex dynamics of forward bias-induced threshold voltage drifts in GaN based MIS-HEMTs by stress/recovery experiments. IEEE Trans. Electron Devices.

[29] Stockman A., Canato E., Tajalli A., Meneghini M., Meneghesso G., Zanoni E., Moens P., Bakeroot B. On the origin of the leakage current in p-gate AlGaN/GaN HEMTs. Proceedings of the 2018 IEEE International Reliability Physics Symposium (IRPS).

